# Perceptions of Mental Health and Wellbeing Following Residential Displacement and Damage from the 2018 St. John River Flood

**DOI:** 10.3390/ijerph16214174

**Published:** 2019-10-29

**Authors:** Julia Woodhall-Melnik, Caitlin Grogan

**Affiliations:** Department of Social Sciences, Faculty of Arts, University of New Brunswick in Saint John, 100 Tucker Park Road, Saint John, NB E2L 4L5, Canada; caitlin.grogan@unb.ca

**Keywords:** mental health, residential damage, residential displacement, coping, climate change, housing

## Abstract

Climate change has spurred an increase in the prevalence and severity of natural disasters. Damage from natural disasters can lead to residential instability, which negatively impacts mental health and wellbeing. However, research on the mental health of residents who are displaced after natural disasters is relatively novel and needs more study. This study investigates experiences of mental health in residents in New Brunswick, Canada, who experienced residential damage and/or displacement during the 2018 spring flood. Lived experiences were studied through focus groups with 20 residents and perceptions of community mental health and wellbeing were captured during key informant interviews with 10 local community leaders. Data collection and analysis employed grounded theory. Findings indicate that those who had residential displacement or damage due to the flooding experienced negative mental health impacts, both during and following the flood. While natural disasters have devastating impacts on mental health, the data also indicate that the communities were positively impacted by a collective and collaborative response to the flood. This paper argues for the utility of communal coping as a concept to describe the experiences of communities following residential damage and/or displacement following natural disasters.

## 1. Introduction

The Lancet Countdown on Climate Change and Health finds that climate change has current health impacts and poses the biggest health risk of the 21^st^ century [1]. Research finds that the impacts of climate change on the physical health of populations are significant and will continue to worsen [1,2]. Physical health concerns arise from the many consequences of climate change, including, but not limited to, exposure to extreme heat [2,3], water contamination [4], air pollution [5,6,7,8], and increased prevalence and spread of infectious disease [9,10,11]. Although the physical health consequences of climate change are well documented, there is less awareness of the mental health implications of the increased magnitude and prevalence of climate-related, natural disasters. 

Experts agree that climate change is associated with the increased magnitude and prevalence of natural disasters [12,13,14]. Climate change-related natural disasters impact mental health and wellbeing [12,15,16,17,18,19,20,21,22]; however, the literature that explores mental health post-disaster is sparse [20] and seldomly focuses on the implications of residential damage and displacement. The prevalence of large-scale natural disasters has increased, which has prompted researchers to call for more empirically based research on the mental health of affected individuals and communities [20]. Further, research indicates that different communities respond to natural disasters in different ways [23,24,25], which suggests a need for additional data from a variety of disaster-prone communities, which have yet to be systematically studied. 

Floods are the most common form of major natural disaster and flood damage recovery periods are often long and strenuous [26]. A systematic review of literature on mental health and floods [26] finds that they can lead to a variety of mental health concerns, such as PTSD, stress, anxiety, and depression, in affected populations. Additional concerns include bereavement and grief, increased substance use, increased intimate partner violence, economic stress and behavioural problems in children [26,27]. Considering the multifaceted nature of mental health following flooding, this paper uses the World Health Organization’s [28] definition: Mental health describes a state of well-being in which an individual realizes his or her own abilities, can cope with the normal stresses of life, can work productively and is able to make a contribution to his or her community (page. 38).

Research that documents the association between mental health and disasters often measures clinical symptomology for Post-Traumatic Stress Disorder (PTSD) [26,27,29,30,31,32,33,34,35,36,37]. The present paper uses a broader definition of mental health, as the goal is not to measure clinical symptomology, but rather to understand the lived experiences and perceptions of individual and community wellbeing following disasters. The singular focus on post-traumatic stress disorder in disaster and mental health research and policy is critiqued as being too narrow in scope [38]. The move towards the use of a broader understanding of mental health has long been supported by health promotion and public health natural disaster research [39,40,41,42]. Broader definitions of mental health, including the one provided by the World Health Organization, have been used by other researchers who study the impacts of natural disasters on human health [38,40].

The World Health Organization’s definition of mental health is prevalent in social determinants of health literature. The social determinants of health are the social, environmental, individual, and economic factors that influence an individual’s health [43]. In Canada, Raphael [43] argues the social determinants of health are: Indigenous status, disability, early life, education, employment and working conditions, food security, health services, gender, housing, income and income distribution, race, social exclusion, the social safety net, unemployment, and job security. The social determinants of health impact individual health outcomes in natural disasters [44].

Early research on mental health and disasters employs stress models to estimate the factors associated with victims’ mental health outcomes [45,46,47,48,49,50,51,52]. Models indicate that a variety of individual demographic factors, life events, mental health history and personal resources impact post-disaster mental health. Individual factors interact with disaster-specific factors, such as severity and residential evacuation or displacement, to predict individuals’ short- and long-term mental health outcomes [46]. More recent evidence confirms that access to resources, individual-level characteristics and disaster-specific factors all play a role in coping and mental health following disasters [53,54]. Those who evacuate their primary residences during disasters often do so because of the severity of the event and they typically experience more damage, chaos, and threats than those who can remain in their homes [55,56,57]. Residential evacuation promotes stress and uncertainty, which are said to lead to poor mental health outcomes [56,58]. Mental health services for residential evacuees are often inadequate and do not provide the long-term support necessary for recovery [58].

As noted above, the prevalence of large-scale disasters has increased and recent literature points to the importance of understanding mental health within the context of disaster response and recovery [15,16,17,18,19,20,21]. However, researchers have demonstrated a need for more research on mental health and climate change [20], which occurs in different community contexts [23,25]. The objective of this paper is to investigate the impact of the 2018 St. John River flood on the mental health and wellbeing of communities who experienced residential damage and/or displacement. 

## 2. Methods 

Data on mental health after a natural disaster are particularly difficult to collect [31,34,59]. Post-disaster researchers often experience ethical, methodological and logistical challenges. For example, populations affected by disasters may be traumatized and vulnerable [59,60]. As such, focus must be placed on data collection that is minimally invasive. One suggestion is to gather data through focus groups, which can provide participants with additional social support and empowerment through conversations with others who have similar or shared experiences [61]. Another tactic involves collecting data on impressions of community well-being from key informants. Interviews with informed community members allow researchers to gather high quality evidence while decreasing the psychological risk and burden to survivors and victims’ family members. In the present study, focus groups with residents and interviews with key informants were conducted. The data collection procedures for the focus groups and key informant interviews are discussed below.

All participants provided informed consent to participate in this study. This study received approval from the Research Ethics Board at the University of New Brunswick in Saint John [file number: 036-2018].

### 2.1. Research Context 

The St. John River, originally referred to in Malisset as the Wolastoq, (We acknowledge that this study took place on the unsurrendered and unceded traditional lands of Wolastoqiyik (Maliseet). At present, efforts have been made by Indigenous community members to restore the name of the St. John River to its original name the Wolastoq. Local Indigenous populations have vast knowledge of the river and its flooding patterns, as such, they have avoided settling too close to the river’s banks and in flood prone areas.) which translates to good and bountiful river, is a vast river that spans northern Maine and western New Brunswick. This research explores the experiences of settler communities and future research should focus on the impacts of flooding on the mental health of Indigenous people who maintain close connections to the river. The river reaches frozen areas in northern New Brunswick, and each spring, thaws during an event that locals call the spring freshet. The present research was conducted within a year of the 2018 flood and within two months of the 2019 freshet, which was predicted to also cause extreme flooding. During the spring freshet, the ice in the north begins to melt and additional water is pushed along the river to drain out into the Bay of Fundy which leads to the Atlantic Ocean. Water levels rise during this period and localized flooding is common. The floods tend to be mild and manageable. However, over the past century, flood levels have occasionally risen to catastrophic levels, which results in significant residential damage. Record breaking floods in New Brunswick that caused an estimated 10 million dollars or more in damages include the floods of 1923, 1973, 1987, 1993, 2008, 2014, 2018 and 2019 [62,63]. These floods caused significant damage to homeowners and resulted in the evacuation of many homeowners from their properties [64]. 

In 2018, the St. John River rose over 6 m in some areas and caused significant damage in low-lying areas of New Brunswick. The Emergency Measures Organization, a provincial branch of government that is responsible for providing emergency response and support, estimates that 2000 to 3000 residents were displaced from their homes during the 2018 flood. In total, 12,947 homes were damaged in the flood [62]. This is significant in a province with only 319,773 private dwellings [63]. Evacuations occurred as power was cut off to homes and as structures were deemed uninhabitable [64].

This study focused on three main communities surrounding the St. John River: Saint John, Kingston Peninsula and Jemseg. While close in location, demographics vary substantially between them. While Saint John is an urban center with a population of 67,575 residents [63], Jemseg and Kingston Peninsula are both rural. Jemseg is a part of the larger Cambridge Parish with a population of 647 residents [63]. The Kingston Peninsula has a population of 2913 [63]. Socioeconomic conditions, such as poverty rates, are similar between these communities [63]. These communities have different access to services; Kingston Peninsula and Jemseg are both unincorporated areas. This means that they are overseen by resident-based town councils and have no formal municipal infrastructure. They have volunteer-based emergency services and receive access to funding and resources for emergency response from the provincial government. Saint John has a formal municipal government with built in emergency infrastructure. Despite these differences, all three communities received extensive support from volunteers. However, one could argue that the role of volunteers is important in the city, whereas it is critical in the unincorporated areas. Our research does not indicate any differences in residents’ perceptions of volunteering between urban and rural areas. Rurality and service provision is a topic to be explored in other papers.

### 2.2. Key Informant Interviews

The key informants in this study were local community leaders and individuals who were involved in or had insider knowledge of disaster relief efforts. To maintain their confidentiality, they are referred to as key informants throughout this manuscript. The researcher developed a list of 16 potential key informants by looking through local newspaper articles on the 2018 floods for influential individuals and by collecting names of local leaders through an internet search. Individuals on this list were sent an email. Those who did not respond were sent a follow-up email two weeks later. Individuals without publicly available email addresses were contacted up to two times via phone. Ten key informants participated in the study and interviews were arranged at times and locations that were mutually convenient.

Participation involved a one-time, semi-structured interview. The researcher posed questions about the participant’s involvement in the community and their perceptions of the response to the flood and community members’ mental health and well-being. These questions are provided in Appendix A. A research assistant attended some of the interviews as a part of her training in qualitative research methods. The interviews ranged from 30 min to two hours in length. All the key informants consented to audio recording of their interviews. The recordings were transcribed for analysis by a transcription service. Interviews were conducted in January, February and March of 2019.

### 2.3. Focus Groups

The research team conducted focus groups in order to reach as many residents as possible in a minimally invasive way. These focus groups were designed to facilitate small, meaningful group conversations about flood experiences and mental health with local community members. The group nature of these focus groups allowed individuals to build on one another’s experiences while they contributed their own perspectives. 

The focus group participants were residents of southern New Brunswick (*n* = 20) who experienced residential displacement and/or damage during the flood of 2018. Only participants over the age of 18 were included in this study. Recruitment was conducted in two ways. First, research assistants hung posters in public locations (e.g., mailboxes and telephone poles) in flooded areas. These posters were also shared on local social media groups, such as those found on Facebook. Second, the researcher contacted local newspapers, radio and TV stations which published or aired calls for participation in the research. Potential participants were asked to email or call the researcher to enroll in the study. Some of the participants contacted other community members who had experienced the flood and invited them to register for focus groups with them.

In total, four focus groups were held and the number of participants in each ranged from four to eight. Participants were grouped based on location and availability. Three focus groups were held on the Kingston Peninsula and one was held in Jemseg. All participants lived within a one-hour drive of each of these locations. The choice of locations was not purposeful; rather, the locations were chose based on participant response. These towns experienced flooding and are centrally located to other areas that also experienced flooding. Each consultation was approximately two hours long and was held at an accessible location such as a community center or local school. Participants were not compensated for their time; however, coffee, tea and light snacks were provided. The focus groups were held in March and April of 2019.

The focus groups began with a registration period. During this time, informed consent was collected and participants filled out a short demographic survey which also asked them an open-ended question on their mental health during and following the flood. The open-ended question was posed to provide those who did not feel comfortable speaking about mental health the opportunity to do so privately. The responses to these questions mirrored the data collected in the focus group notes and are provided in Appendix B. After participants completed the paperwork, they sat in a circle and the focus group began with the researcher’s request for people to talk about their experiences with residential damage or displacement. The focus groups were semi-structured around four central themes and the conversation often flowed with little active facilitation. The facilitator steered the conversation toward one of the central themes when the conversation went off topic or when there were lulls in discussion. At the end of each focus group, the facilitator reviewed her notes on each of the four themes and additional questions were posed when clarification or elaboration on themes were needed. The central themes were residential displacement and damage, the impacts on mental health and wellbeing, social capital and recovery. Two research assistants were present at each of the four focus groups and the Principal Investigator [JWM] facilitated each group. The researcher and research assistants each took notes on each focus group. Each focus group culminated with three sets of notes. The final data products were 12 focus group notes. The notetaking procedure allowed for researcher triangulation and provided more complete coverage of the conversations.

### 2.4. Analysis

The data collection employed grounded theory. The researcher and research assistants debriefed on emergent themes after each focus group. The researcher reviewed her research notes and audio files after each key informant interview. During these debriefs and reviews, emerging themes were discussed or noted, and interview guides were amended to include emerging foci. After data collection was complete, the research team followed Burnard’s [65] methodology for coding qualitative transcripts. The analysis was organized using MS Excel.

The research team began by analyzing the key informant interview transcripts. Five transcripts were randomly selected for inclusion in the open coding process. The analysis team consisted of the researcher and two research assistants. Each team member was randomly assigned three of the five transcripts to review. This allowed the analysis team to confirm consistency and allowed the team greater opportunity to discuss findings and emergent themes. The analysis team met to discuss themes that emerged in the five transcripts and developed a preliminary list of themes and sub-themes. The team then assigned definitions to each theme and sub-theme which were used to guide the analysis. These themes were used as a preliminary codebook. The analysis team used the preliminary codebook to analyze all 10 transcripts. Changes to the codebook were made when deemed appropriate and with consensus of the analysis team. There were 27 themes in total.

The analysis team repeated this coding procedure with typed notes from the focus groups. The team began with five randomly selected focus group notes. Each team member reviewed and assigned preliminary codes to three of the five notes. The analysis team then met to discuss the initial reviews and to construct a preliminary codebook. The analysis team used the preliminary codebook to analyze all 12 sets of focus group notes. Changes to the codebook were made when appropriate and with team consensus. There were 32 themes in total.

The researcher J.W.M. and one research assistant C.G. who are the authors of this paper, reviewed each of the themes from the coding of the focus groups and key informant interviews and performed focused coding on each of the themes that aligned with the main objective of this paper. The focus group themes included for focused coding were: negative impacts on mental health; positive impacts on mental health; isolation; loss; and coping. The key informant interview themes included were community emotions and self-reliance. The findings from this analysis are presented below.

## 3. Findings

The analysis revealed that mental health during and following flooding is complex and intricately intertwined with the lives and actions of community members. The data indicate that those who experience residential damage or displacement due to flooding experience negative impacts on mental health both during and following flooding. These impacts stem from exhaustion, stress, anxiety, worry and uncertainty. At the same time, participants noted feeling connected to their communities, grateful and thankful for the support and help they received. 

### 3.1. “It’s Going to be a New Normal”: Mental Health During and Following the Flood

The key informants and residents used a variety of terms to describe mental health and wellbeing during and following the flood. Stress was discussed frequently by the key informants and the residents. The term stress was used in multi-faceted ways to discuss concerns surrounding finances, evacuations and the rebuilding and recovery processes. One of the key informants stated:

You think of the biggest thing you’ll buy in your life probably you know, with the exception of some people that have lots of wealth and when danger comes to your home regardless of what form it’s in then the home owner becomes very anxious and you know, people have families and in our society today everybody has to try to make ends meet. There’s lots of stress out there involved in that…people with families, young families, doesn’t matter the age. 

This statement was echoed in the focus groups when residents discussed the challenges they experienced while trying to access financial assistance for home repairs. One of the focus group participants who had not yet returned to her home spoke of her inability to access government assistance for repairs. She was uncertain about what her future held, as she was unable to pay for her home repairs. She said that this caused her a large amount of stress and had resulted in relationship problems between her and her partner. Other individuals described the process of filing paperwork and claims for compensation with insurance companies and the government as stressful and confusing. 

Stress and guilt were used to describe the evacuation process for residents who left their homes and communities. One key informant stated:

*“There was a lot of physical energy that was exerted in trying to preserve their home, their property…the stress of some giving up, losing that fight and not keeping the waters at bay was difficult. Knowing their losses would probably be greater than what government assistance was going to be. I’m sure that it was a very difficult and stressful time for many.”*

This key informant noted that evacuating after trying to preserve one’s home was a stressful experience for residents. However, not all residents felt this way. During the focus groups, some described the stress of leaving their homes and the worry experienced when they chose to stay and felt isolated; however, one resident described giving up as cathartic. She noted that she was able to stop worrying and fighting and that she moved on to help her neighbours try to keep the water out of their house. Another participant described the experience of giving up as guilt inducing. After her power was cut off, she could no longer run her generators and pumps and decided to evacuate. She felt relieved that there was nothing more she could do but also felt guilty for leaving her neighbours and felt like she was missing out on a key community event.

The key informants and the residents described the physical and mental exhaustion that was felt during the flood. One resident discussed her experience of checking on her neighbour during the flood. She said that her neighour was sitting in a darkroom with bags under her eyes. She looked like she had not slept in days and she was surrounded by ashtrays full of cigarette butts and a half full bottle of liquor. She was watching the news reports on the flood. Exhaustion was also discussed by key informants. For example, one stated:

*“Well, I don’t know about the long-term, but I think that short-term it certainly affects people to the point where they don’t sleep properly, there overwhelmed. They’re trying to get things back to normal, but it’s going to be a new normal for a lot of people. It’s not going to be the old ways. So, I think for the short-term, for sure it has an effect on people mentally and they’re just exhausted. ”*

The amount of physical and mental energy put into trying to keep water out of homes and later into clean up and rebuilding was referred to as “exhausting” by key informants and residents. During the flood, the impacts of not sleeping for days were felt. The lack of sleep increased stress levels and the residents attributed a lack of sleep to both physical and mental exhaustion. One key informant noted that the freshet is normally three or four days and that the residents experienced 11 days of flooding where they were essentially trapped in their homes and fighting to keep their belongings dry day and night. This meant that residents stayed awake to move belongings to higher areas in their homes, woke frequently or stayed up to monitor pumps and flood projections, and spent countless hours on flood mitigation efforts to keep the water out of their homes. One of the key informants noted that residents have three days following a flood to remove wet materials and items from home before mold begins to grow. Once the water receded, residents, already physically and mentally exhausted from the flood, began the race against the clock to get rid of waterlogged insulation, carpets, mattresses, and other household items.

The communities were devastated by loss during the flood and this had an impact on mental health. One of the key informants described the loss of community felt by some of the residents of nearby cottages:

*“[H]uge impact for cottagers because of course, communities were wiped out and I think we lose sight of that, because those cottages were built on nothing, right. And so, like I talked to one cottager in her little community…only two cottages survived, and so the two people with the cottages that didn’t get destroyed had survivors’ guilt…And so entire communities were wiped out by that flood. ”*

The community impacts were also felt in one town where their local community centre was demolished because of the flood. The focus group participants described the important role that the centre played as a hub for the local community. The centre hosted local weddings, events and community meetings. They used the term ‘devastated’ to describe their feelings when they found out that funding was not available to rebuild the community centre. They noted that they were brought together as a community during the flood and had no place to gather afterwards.

The fear and anxiety associated with being isolated was a topic discussed by the focus group participants. This was not discussed by the key informants. To get to the Kingston Peninsula, cars can take one of three year-round ferries or one access road from the town of Hampton. During the flood, all but one of the ferries to the Kingston Peninsula were shut down due to high water levels, which made commuting an extremely long and onerous process. A nearby community, Darlings Island, is only accessible via one access bridge. During the flood, the bridge to Darlings Island had flooded over and the only way off the island was by boat. A volunteer ran a boat service to and from the island during the 2018 flood to assist stranded residents; however, this was the only option available to leave and get back onto the island. During the focus groups, participants noted that residents of Darlings Island are moving because they fear being cut off from the mainland. People were particularly concerned about getting to work and accessing medical treatment in an emergency. One resident noted that people who live on the island and work as medical professionals at the nearby regional hospital, one of the largest employers in the area, leave the island during flooding to ensure that they can get to work. Residents who lived in areas that were cut off from neighbouring towns reported feeling isolated and anxious. These residents experienced significant life disruptions.

The focus groups took place in the two months leading up to the 2019 freshet. The residents discussed their anxiety as they planned and waited for what the 2019 season would bring. One participant noted that his blood pressure spiked every year around the time of the flood and returned to normal after the spring thaw had passed. Two of the 20 focus group participants discussed the impacts of the flood on their relationships. One resident noted that her husband copes differently with floods than she does. She noted that he is calmer and more apt to seek avenues to repair their home, whereas she does not want to continue to live in a flood zone. She said that differences in how they cope with floods has had taken a toll on their marriage and that they frequently fought. One of the participants also noted that her and her boyfriend, who were not yet back in their home, had experienced increased tension in their relationship since the flood. She said that the extra stress, uncertainty about their home, and the lack of permanence in their living arrangement caused tension and conflict in their relationship. Men did not talk about relationship suffering in the present study. As men are socialized to avoid disclosure and be less emotionally expressive, they may be less likely to discuss relationship problems in a focus group setting.

### 3.2. “When the Chips are Down, People Come out and Help”: Community Responses to Flooding

The focus group participants and the key informants all spoke about the important role of the local community of residents in their discussions of mental health and wellbeing. During the flood, there was a large sandbagging initiative that was spurred by local firehalls, first responders and the Emergency Measures Organization. This initiative attracted many volunteers from across Southern New Brunswick. One of the key informants described the sandbagging:

*“So, with the sandbagging, what you could see is that all the different communities all down the river valley is that that was a coping mechanism to handle the stress that was involved with the event. What can I do? What can I do? What can I do? I’m just going to go fill sandbags, right?”*

The focus group participants also discussed the sandbagging. They noted that people helped others regardless of whether they knew them or not and that the act of helping seemed to calm people who appeared antsy or nervous. They stated that it provided them with a sense of control over the events that were taking place. The participants noted that they felt like they were not able to keep flood waters at bay and this was frustrating as they waters were outside of their control. They did note that one thing that they could do was participate in sandbagging initiatives. This is something they felt they had control over. 

The focus group participants described witnessing the aid they received from fellow community members as a “positive, unbelievable” experience. One participant said that she “watched people come together and it really put into perspective what’s important.” Others felt reassured that they knew that they could count on their neighbours and some participants said they felt “fortunate” and “blessed.” One of the key informants said:

*“I think one of the things that was good for communities, for individuals that were struggling, and for those that kind of felt helpless was having sandbag stations. I know it sounds really simple, but you know, people wanted to help. And that was really the easiest way to help….and kids were able to help that way….it really did give a sense of being able to help, I think. ”*

This key informant highlights the positive contribution of the community as a unified group that came together to support one another. When discussing community mobilization during the flood, another key informant referred to people’s willingness to help others as a “maritime thing.” The sentiment that the aid and assistance was positive for communities and victims of flooding was expressed in every focus group and interview. The idea that the community assistance was unique to New Brunswick and the maritime region of Canada was presented by many study participants. 

The Key Informants and the focus group participants discussed the role of children in filling the sandbags. During the flood, volunteers came from all age groups. One Key Informant said:

*“During the flood you just saw community groups opening up the doors to get sand, fill bags to give to people whose houses were flooding. The Grand Bay primary school had kids playing in sand, putting them in bags and like getting people ready for the flood.”*

Some of the focus group participants described seeing mothers with their preschool aged children filling sandbags. These participants thought it was very positive and encouraging to see and they also thought it was a great opportunity to teach the kids about being a good community member and citizen. 

There were some lasting impacts of the flood on community building. In Jemseg, the community banded together to host a flood preparedness weekend at a local community hall. This weekend focused on planning for the upcoming spring freshet in 2019. One of the key informants discussed this:

*“I’m seeing people really take action now and at a community level…there is the Village of Jemseg which is a very small—my grandmother would say it is huge, but I’m like no, it’s really not. [The preparedness] is coming down to a small local level. People there are taking that next step [to prepare for next year]. ”*

Following the Freshet, this small community banded together to talk about flood mitigation efforts and preparedness for the following year. These organic efforts arose from the community engaging with one another during the flood in 2018.

Although community involvement was viewed as a positive coping mechanism by all the study participants, there was one focus group participant who concurrently described additional negative impacts of community cohesion and coping. This participant saw the community network as anxiety provoking as everyone was going through trauma together and collective discussions of trauma, although intended to be supportive, caused this participant to take on others’ anxiety. A few of the focus group participants noted feeling guilty for not being able to participate in the community response and help their fellow neighbours while trying to save their own houses. Another participant described the isolation that comes from not having a community nearby. She stated that when you are isolated in an area without a large community, you feel as if you are isolated and left to cope on your own. This participant was relatively new to the area and lived in an area that was only inhabited by two cottages and her own home. She was encouraged by other people’s stories of community building; however, felt that a lack of community can be very damaging when others are relying on their neighbours for support. 

## 4. Discussion

The resident focus group participants’ and key informants’ experiences indicate that the flood of 2018 had impacts on their mental health and wellbeing. The key informants often focused on the financial stress experienced by residents. Communities that experience financial disadvantage are more susceptible to the negative impacts of natural disasters [66,67]. Socioeconomic disadvantage contributes to increased risk of poor mental health and wellbeing during and following natural disasters. Disadvantage is also associated with social isolation [41]. In New Brunswick, individuals who experienced damage to their homes experienced some form of financial loss. When repairs and losses are insured, residents are often required to pay for expenses up front and wait for reimbursements from government bodies and insurance companies. This is problematic as not all households have the equity or available capital to cover repair expenses and wait for reimbursements. This was described by one of the key informants who noted that many residents in flood areas are not economically wealthy and stress arose from the financial damage caused to homes.

The residents found the flood recovery process to be stressful, onerous and confusing. They were not always clear about where to get help and felt that more guidance was needed to assist them as they made choices about rebuilding, relocating, or using flood mitigation tactics (e.g., raising a house). For two participants, this led to domestic conflict which is found to be a consequence of natural disasters [68,69,70,71]. The residents’ experiences indicate that more post-flood support and information should be provided to residents. Additionally, interventions to reduce family conflict and domestic violence following natural disasters should be developed and targeted to households who have experienced loss and stress.

The residents described the fear and isolation that they felt during the flood. This fear continued to impact their lives as they experienced uncertainty and anxiety leading up to the next flood season. Isolation and perceived isolation are found to impact well-being [41]. In the 2019 flood, the provincial government of New Brunswick provided boat shuttle service to the residents of Darlings’ Island to reduce the physical isolation residents of the island. Whether the boat shuttles impacted feelings of isolation has yet to be determined; however, a follow-up student of the St. John River flood of 2019 is currently underway.

Despite the negative impacts of residential damage and/or displacement from the flood on the mental health of residents, our data indicate that the flood had some positive impacts on local communities. Disaster researchers argue that exclusive focus on negative mental health impacts provides incomplete understandings of experiences [First]. Studies indicate that community engagement can provide positive experiences of mental health following disasters [72,73,74]. The present study confirms these findings. All study participants discussed the positive impacts of community efforts during the flood response. Recently, two terms emerged to describe the impacts of community engagement following disasters: communal coping [48] and post-traumatic growth [72,73].

Post-traumatic growth, a concept coined by Tedeschi and Calhoun [75], is frequently used by researchers to describe positive psychological changes in individuals who experience significant traumas and adversity [76,77]. These psychological fluctuations promote positive changes in individuals who experience post-traumatic growth and overcome PTSD. Hence, the presence and measurement of PTSD symptomology is fundamental to the application of post-traumatic growth, wherein growth is attributed to the process of overcoming trauma from struggle [78]. It is said that these individuals who experience post-traumatic growth can function at a higher level than they could prior to experiencing traumatic events [79]. This higher function may manifest as better relationships with others, spiritual growth, more positive outlooks on life, a reappraisal of priorities and belief in positive life opportunities, and increased self-confidence and self-perceived strength [75,76,80]. The term is frequently applied to individuals who have diagnosed with major physical illnesses [76,81,82,83,84] or who have lived through natural disasters [85,86,87,88].

Natural disasters produce environments where adversity is likely to be tackled through communal efforts [82] This was witnessed New Brunswick communities during the 2018 flood, where residents joined large sandbagging efforts in attempt to save homes and infrastructure. Affifi et al. [55] argue that communal action can be directly stated through group members’ messages and words or indirectly displayed through community building actions (e.g., participation in sandbagging and clean-up efforts). Communal coping has been offered as a term to describe communal efforts [55]. Communal coping refers to the process of communities coming together to become cohesive, resilient groups who view uncertainty and stress as solvable through communal and collective action. This was apparent in the group from Jemseg who created a disaster mitigation workshop to prepare affected residents for future floods. One could argue that the stress and uncertainty associated with the possibility of another large flood was mitigated through this workshop effort.

In their study of residents’ mental health following Hurricane Ike, Richardson and Maninger [74] find that residents engage in three communal coping activities: mutuality; co-construction of a community narrative; and problem-centred communal coping. Mutuality involves a community’s recognition that they are all impacted by a shared problem and sharing stories with one another to cope. The focus group participants and key informants noted that the community did band together to support one another. Part of this support was providing resources and information. Many residents discussed this as positive; however, hearing neighbours’ experiences was anxiety provoking for one resident who was worried about saving her own home.

Problem-centred coping involves providing material assistance to others in the community and sharing information and experiences with help-seeking. This assists residents who are in search of resources; however, it can also cause negative emotions for those who recognize that they have received less help than their neighbours [74]. Many of the residents benefited from the material and physical resources of others who participated in the sandbagging and clean-up efforts. They also benefitted from the advice of neighbours. However, those who did not have communities to rely on for support, assistance and advice, such as the young woman who had recently moved to the area, or those who evacuated and left their communities during the flood, felt isolated and guilty. This suggests that greater social, emotional and physical support may need to be provided to individuals who are displaced from or are not connected with local communities. Further, practitioners who work with residents who experience residential damage and/or displacement may benefit from awareness of the propensity of communities to cope in collective ways and the benefits and emotional struggles that may ensue for those who are isolated.

Communities that go through communal coping are said to co-construct narratives which describe their communities in romantic fashions. In these narratives, people pull together and collectively work hard with little outside or governmental assistance. These narratives focus on moving forward collectively as a community and describe a new recognition of self and community through recovery processes. In these narratives, communities are seen as special or unique for their abilities to act collectively [74]. Other regions that have experienced natural disasters have devised similar narratives [74,89]. Co-constructed narratives were highly apparent in the present study. The participants all discussed the uniqueness of the community as a tight knit group of residents who were able to pull together to live through and rebuild after the flood. Community cohesion was seen as something that is unique to eastern Canada and New Brunswick, especially by the key informant who described it as “Maritime thing.” Many residents and key informants noted that the flooding make them aware of the true value of their communities and some noted that they had increased appreciation and felt lucky after witnessing the communal response to the flood. 

Although post-traumatic growth is used more frequently by disaster researchers to describe positive mental health impacts, communal coping, as a process, rather than an outcome, may be better suited to qualitative understandings of collective action. If PTSD persists over a prolonged period, communal coping may be best to understand the coping that occurs soon after disaster events. In fact, research has found that communal coping is an antecedent to post-traumatic growth [90]. Communal coping may also be best to understand the experiences of those who do not have PTSD symptomology. In order to experience post-traumatic growth, one must experience PTSD. This is highlighted in the work of Joseph et al. [91,92] wherein they describe PTSD as the “engine” of post-traumatic growth. PTSD is the most studied psychosocial impact associated with disasters [24]. Thus, the concept of post-traumatic growth may be beneficial to quantitative, epidemiological research on mental health following disasters. However, a variety of clinical symptoms must be present in order to illustrate that someone possesses the pathology to receive a diagnosis or illustrate clinical probability of PTSD. Further, PTSD is an individual diagnosis and cannot describe the mental health or well-being of a community.

In recent years, literature on disasters and mental health has moved away from a focus on psychopathology and requiring clinical PTSD to evaluate community wellbeing does not follow that shift. Traumatic events are defined as exposure to threatened death, serious injury or sexual violence according to the DSM-5. This definition itself excludes many individuals impacted by natural disasters, as some were never in any physical danger. The term post-traumatic growth has been used in discussions of community resiliency and recovery efforts see: [93]; however, the present authors argue that it is best suited to discussions of individuals with PTSD symptomology who experience individual changes in outlook and functioning. Rather, the experiences of residents along the St. John River during the 2018 spring flood indicate that communal coping may be more useful for understanding communities’ experiences of mental health, wellbeing and positive collaboration following natural disasters. The collective responses that are integral to communal coping, reduce the impact of trauma by fostering individual post-traumatic growth and overall social wellbeing of those impacted by natural disasters [89]. The present study also finds this, as residents viewed communities’ responses as positive. The definition of communal coping encompasses the changes of the entire community, beyond those with PTSD symptomology, allowing for a broader understanding of growth following natural disasters.

## 5. Conclusions 

This study was largely exploratory and aimed to understand the presence or lack thereof, of mental health and well-being impacts of floods in Eastern Canada. In 2019, the St. John River once again rose to high levels and, once again, caused major damage and destruction to nearby communities. A follow-up study on the 2019 flood is currently underway to specifically investigate community coping strategies and explore residents’ suggestions for targeted interventions to improve the health and wellbeing of disaster affected communities. This paper focuses on the St. John River flood; however, it is important to note that other communities have and continue to experience flooding. It is imperative that researchers continue research in this area. For example, quantitative studies of a variety of different locations could be conducted to create an evidence base that is generalizable to larger populations of persons who experience residential damage and displacement due to flooding and other natural disasters. As natural disasters continue to grow in intensity and frequency, research on the environment and mental health should expand to inform academics and practitioners about the risks and community adaptations that result from climate change.

Residents who live along the St. John River are not strangers to floods. However, the intensity, duration and loss caused by spring floods has increased in recent years. The emotional toll that this takes on residents and communities is important to understand, as climate scientists predict that Eastern Canada will continue to experience devastating floods. The findings from the study of the 2018 flood found that residents’ mental health was negatively impacted by the residential damage and displacement caused by the flood. However, community involvement and collective response to the flood emerged as positive results of the flood. This can be explained using the concept of communal coping. As communities continue to respond to disasters, it is important that we test coping frameworks to learn how practitioners, responders, and academics can intervene and provide support to persons who are displaced or experience significant damage to their homes.

## Appendix A. Interview Questions for Key Informants

(1) I would like to know a bit about your involvement with the local community. How do you normally interact with the community? What is your role?
(2) Were you involved with disaster relief efforts in this community? If so, can you tell me a bit about how you were involved? What was this experience like?
(3) How do you think that the flood impacted the local community? Did it have an effect on mental health and wellbeing? Can you elaborate?
(4) What types of supports were provided to residents? Where did these supports come from?
(5) Is there anything you think that community members needed during the flood that they did not have access to?
(6) If a disaster like this were to happen again, what would you keep the same about the response? What would you change?
(7) Is there anything else that you would like to share that you think I should know?

## Appendix B. Focus Group Participants’ Written Responses on their Mental Health and Wellbeing Following the Flood of 2018

**No Major Impacts on Mental Health and Well-Being Reported**
The impact on us was relatively small and we were financially able to repair the damage.
Not Applicable
No, I didn’t really have to deal with the daily issues. My mother and her husband have been affected. They worry about selling the house, they worry about the next flood. They are disheartened that the government does not understand their situation.
**Responses Clearly Indicated Impacts on Mental Health and Well-being**
Yes. We live on an island, our road was barricaded, instead of leaving we were stuck for 15 days. My property was the departure/arrival space for boaters. There was so much going on, no privacy and worrying if anything happened to any member of my family how we would get to the mainland for help.
The flood was very stressful and a lot of hard work. We didn’t have money to fix anything and we are still suffering. Our house got flooded in the basement and we only got enough money to gut it and get half of the walls fixed. Also, we haven’t even touched the camp yet (we have a camp on our property). 6000 won’t even cover the cost to clean out what is in there. We also have 3 kids that live with us about half of the time. Our house was a mess for a while, we had to bring everything upstairs. That’s only half of it. We sand bagged to prevent the water from hitting our house but the water went through the ground.
Yes- makes me feel very unsafe and uncertain in my own home every year. Very uncertain if I want to take major construction to raise the house but I believe it will happen again. Frustrating because my husband and I don’t see things the same way- has caused some short tempers.
Yes, traumatic, depression.
Yes, anxiety about impact of flood. Stress + the isolation of being partly cut off.
Yes- did not sleep for 9 days, took from May 2-July to finish damage, dealing with government payment plan.
Yes, major stress throughout. Financial burden in having to finance repairs/rebuild; disruption to work life; disruption to kids’ lives.
Yes- It took a huge amount of physical labour to repair our property. The high wind day caused a huge amount of fear + anxiety. The anxiety stayed with me for many months after. My home, which should be my place of safety, no longer feels safe.
Yes. Mental health- not knowing the extent of damages was extremely stressful. It was a disaster in slow motion. Wellbeing- We were without water + electricity for one month. Every day was challenging, but the kindness and support we received was overwhelming... bitter sweet experience.
I was severely affected by the 2018 flood because it was the second time since the 08 flood. The effects are constantly on my mind especially around early March and April. I can only liken it to PTSD.
It was stressful watching our friends/neighbours cottages being torn apart behind our house and not being able to help and then to see the terrible mess in the fields after. I went to help clean up and could only stay for an hour as it was overwhelming. Some of our friends’ cottages were over 80 years old and had built them and they were deeply affected- have [moved] into trailers.
Yes, it did. My daughter-in-law + son who live on the river’s edge were for many days on the verge of having to vacate their home. With no heat or electricity, I was worried for their health + wellbeing. My brother-in-law and sister-in-law who live 30 miles down the river from our village were flooded out + were not able to stay in their home. As they are in their late 70s it caused great anguish for my husband and I for their health + wellbeing. My brother-in-law’s health has been heavily affected by the flood and its resulting turmoil.
Yes, it has been stressful to cope with, to organize the repairs, and apply for compensation.
It most certainly did. I have never experienced so much stress. The flood has had a detrimental impact on my health, finances, and relationship. Both my partner and I have suffered from depression and have endured many conflicts whilst attempting to recover from this disaster. I have lost over 40lbs since the flood and am still dealing with the stresses of being displaced and trying to manage finances.
It did. I donated my time to helping the residence of Darlings Island coming on and off the island. Seeing the people trying to get to work, school, grocery store, etc. became an impossibility. I felt I had to do my part to help. It was a good, surreal, feeling being able to help those who needed it in their time off especially where other agencies that had the resources to help did not.
I hesitate to say “impact” on my mental health although the extremely long waits at the Gondola Pt Ferry were frustrating as I felt guilty because I couldn’t help others. I had to leave my home at 5:15 each morning and often didn’t arrive home until 7:30 or 8pm. I have a parent in their own home with dementia and I visit 2-3 times per week so my wait times at the ferry were even longer. I knew of many who could have used my help however I was not able. I felt very badly for that. It was heartbreaking to know others could have been helped by able-bodied folks like me.
Yes- it was a short period of time that you were aware you could lose everything/ if you didn’t act fast. You had to “save yourself” as well as help your neighbour- it was intense. I could go on but I feel that sums it up. The feelings of being torn to help + help yourself. The witnessing of community coming together- it was a learning curve in such a vast range of aspects of people/land/water +how you overcome/surrender to mother nature. I feel it was an incredible experience to have lived through it.
